# Hormonal exposures and the risk of intracranial meningioma in women: a population-based case-control study

**DOI:** 10.1186/1471-2407-6-152

**Published:** 2006-06-07

**Authors:** Brian Custer, WT Longstreth, Leslie E Phillips, Thomas D Koepsell, Gerald Van Belle

**Affiliations:** 1 Department of Epidemiology, School of Public Health and Community Medicine, University of Washington, Seattle, WA, USA; 2 Pharmaceutical Outcomes Research and Policy Program, School of Pharmacy, University of Washington, Seattle, WA, USA; 3 Department of Neurology, School of Medicine, University of Washington, Seattle, WA, USA; 4 Department of Health Services, School of Public Health and Community Medicine, University of Washington, Seattle, WA, USA; 5 Department of Environmental Health, School of Public Health and Community Medicine, University of Washington, Seattle, WA, USA; 6 Department of Biostatistics, School of Public Health and Community Medicine, University of Washington, Seattle, WA, USA

## Abstract

**Background:**

The role of exogenous hormone exposures in the development of meningioma is unclear, but these exposures have been proposed as one hypothesis to explain the over-abundance of such tumors in women.

**Methods:**

The association between oral contraception (OC) or hormone replacement therapy (HRT) and intracranial meningioma in women was investigated using a population-based, matched case-control study. Exposures for 143 cases and 286 controls matched on age within five years were obtained by interview. Diagnoses were confirmed histopathologically and estrogen and progesterone receptor assays conducted.

**Results:**

Although risk of meningioma appeared modestly elevated in past OC users (OR = 1.5, 95% CI 0.8 – 2.7), and in current users (OR = 2.5, 95% CI 0.5 – 12.6), the confidence intervals were wide. No significant association between meningioma risk and duration of OC use was found. Likewise, risk of meningioma was only weakly associated with past use of HRT (OR = 0.7, 95% CI 0.4 – 1.3), and not at all with current use of HRT (OR = 1.0, 95% CI 0.5 – 2.2). Of 142 available specimens, 2 (1%) expressed estrogen receptors, whereas 130 (92%) expressed progesterone receptors (PR). OC use was associated with increased risk of a meningioma expressing less rather than more PR (OR = 3.2, 95% CI 1.3 – 8.0). Overall, in post menopausal women, HRT use appeared to confer a non-significant protective effect, and was not associated with low or high PR expressing meningiomas.

**Conclusion:**

This study found little evidence of associations between meningioma and exogenous hormone exposures in women but did suggest that some hormonal exposures may influence tumor biology in those women who develop meningioma.

## Background

Meningiomas occur more frequently in women than men and have the highest incidence in the fifth and sixth decades of life[1,2]. Consistent with data from other studies in North America and Europe, we have previously reported the incidence of intracranial meningioma in western Washington State to be 3.2 per 100,000 person-years, with women comprising 69% of the meningioma diagnoses and the highest age- and gender-specific incidence of over 7 per 100,000 person-years in women 60 years or older[3].

Research has focused on the similarities and risk factors common to meningioma and other neoplasms in addition to other mechanisms. In particular, meningioma may be associated with breast carcinoma[4,5] but this association may not be a direct link and instead may rely on the common predispositions of gender, age, and reproductive factors. Hormonal exposures have a suspected role in the etiology of both neoplasms[6]. The Women's Health Initiative (WHI) study, a large randomized trial, found an increased risk for breast carcinoma in post-menopausal women using opposed hormone replacement therapy (estrogen and progestin)[7]. In a separate WHI analysis, results of unopposed estrogen exposure suggest a non-significant reduction in the risk of breast carcinoma[8]. Observational studies examining hormone replacement therapy and breast carcinoma have reported results generally in agreement with the randomized trials [9-11]. Evidence for an association between oral contraception and breast carcinoma remains equivocal[9,12-16]. A recent report from a case-control study found few significant associations between reproductive or hormonal factors and the risk of meningioma[17]. However, results from the Nurses' Health Study cohort, suggest the risk of meningioma is increased among women exposed to either endogenous or exogenous sex hormones[18]. As part of a case-control study designed to investigate putative risk factors for intracranial meningioma, we evaluated the association between intracranial meningioma and sex hormone exposures, and in relation to hormone receptor expression in the tumors.

## Methods

We conducted a population-based, matched case-control study of risk factors for intracranial meningioma, as detailed previously[3,19]. Institutional Review Boards at the University of Washington and the Fred Hutchinson Cancer Research Center approved the study. All interviewed subjects provided signed, informed consent before study participation. We identified cases with incident intracranial meningioma, histologically confirmed during life between January 1, 1995 and June 30, 1998, using the National Cancer Institute's Surveillance, Epidemiology, and End Results program for King, Pierce and Snohomish counties of western Washington State, which together had a population of nearly 2.8 million at the mid-point of the study. Using random-digit dialing or Medicare eligibility lists, we recruited two controls for each case matched on gender, age within 5 years, and county of residence. The participation proportion for each method of control recruitment was calculated as a percentage based on the number of participants divided by the number of subjects screened and found eligible using that recruitment method. All cases and controls were 18 years or older.

Exposure histories were obtained from all enrolled participants through a structured in-person interview and questionnaire that focused on the entire history of exposures before the reference date. Reference date was the date of the case's surgery that yielded histological confirmation and was the same for the case's matched controls. Seven of the 143 female cases identified for this study were unable to complete the interview due to disability or death. Proxy interviews of a spouse or next-of-kin were conducted for these cases, but were not conducted for the matched controls. For reproductive and medical history in women, we specifically asked about factors such as age at menarche, pregnancy, breastfeeding, and menstrual status in addition to other exposures common to both men and women. For use of oral contraceptives and hormone replacement therapy, we inquired about the type of prescription using visual prompts, the calendar time of use, and duration of use for any hormone therapy. Interview responses on the type of oral contraceptive preparation used were often incomplete, and so we were unable to examine the influence of the type of preparation on the risk of meningioma.

For all 143 cases, we confirmed the meningioma diagnosis by requesting tumor specimens and pathology reports from the resecting hospital. The study neuropathologist confirmed the diagnosis of meningioma. In addition, specimens were submitted to Phenopath Laboratories, Seattle, Washington, for estrogen and progesterone receptor analysis. New tumor tissue slices were prepared from specimens embedded in paraffin, pretreated in citrate buffer pH 6.0 in a steamer for 30 minutes, and placed on an autostainer. For estrogen receptor analysis, antibody clone 1D4 was used (Dako Corporation, Carpentaria, CA, USA). For progesterone receptor analysis, antibody clone 88 was used (BioGenex, San Ramon, CA, USA). Detection was performed using the labeled streptavidin-biotin system (LSAB+) with horseradish peroxidase followed by 3,3'-diaminobenzidine chromogen (DAB+). Receptor assays were successful in all but one case.

### Analysis

We used conditional logistic regression to measure the association between each exposure and intracranial meningioma. For all analyses, we used never-exposed or no exposure as the referent category. Results are reported as odds ratios (OR) with 95% confidence intervals (CI). Where appropriate, tests for linear trend were conducted. Multiplicative interactions were assessed using the likelihood ratio (LR) test. Given their slow growth, most meningiomas have probably been present for a decade or more by the time a diagnosis is made[20]. Therefore each analysis was repeated for exposures beginning at least 10 years before the reference date. We also compared the conditional logistic regression results to unconditional results of models with the same variables. Because the results for these comparisons were not substantially different we elected to report the results using conditional logistic regression as it was the originally proposed methodology for the study. In exploratory analyses, we used polytomous logistic regression to compare hormone exposure categories for controls to cases with tumors that had abundant hormone receptor expression and to cases with tumors that had little or no hormone receptor expression. The odds ratios for these analyses measure the association between exposure and case-control status, examined separately according to the two case groups defined by receptor status rather than between exposure and receptor status among cases only. For both conditional logistic regression and the exploratory polytomous regression, we assessed whether other variables were potential confounders, such as age, education, smoking history, alcohol consumption, body mass index, age at menarche, and parity. When necessary in stratified analyses, we used unconditional logistic regression in order to prevent the loss of data for case-control groups that were concordant on the specific exposures of interest. Analyses were conducted using Stata (version 9, Stata Corporation, College Station, TX, USA).

## Results

Although the 143 cases and 286 matched controls were similar on age, race and marital status, controls were more educated than cases (Table 1). Because education was a confounder, we included it in all multivariable models as a categorical variable: high school or less, some college or trade school, college graduate. Also, because on average cases were 0.9 years older than the first matched control and 1.6 years older than the second matched control, we included age in all multivariable models as a continuous variable to address residual confounding. Case participation was 84%. For controls identified through random digit dialing 55% participated, and for controls identified using Medicare eligibility 67% participated. On average cases were interviewed 410 days after the reference date and controls were interviewed 839 days after the reference date.

### Oral contraception

With never-users of oral contraceptives as the referent group, the odds ratios for past users or current users on the reference date were both greater than 1.0 but not significantly so (Table 2). A graded effect (linear trend) with years of use or initiation of use relative to the reference date was not apparent. Tests for interaction did not reveal significant interactions between oral contraceptive use and pregnancy, number of pregnancies, number of live births, body mass index, hormone replacement therapy, or breastfeeding.

### Hormone replacement therapy

Analysis of the association between hormone replacement therapy and meningioma was restricted to postmenopausal women. Two hundred and seventy three women (101 cases, 172 controls) reported they were no longer menstruating on the corresponding reference date for each matched group. Of these women, 63 (62%) cases and 114 (66%) controls reported hormone replacement therapy use on or before the reference date.

We found no statistically significant association between hormone replacement therapy and meningioma. With never users of hormone replacement therapy as the referent group, the odds ratio for women reporting past use was lower than 1.0 but not significantly so (Table 3). Current use on the reference date was not associated with meningioma. A trend with increased years of use or initiation of use relative to the reference date was not apparent. Odds ratios for the type of hormone replacement therapy did not indicate a significantly increased risk of meningioma for either opposed or unopposed estrogen. Tests for interaction did not reveal significant interactions between hormone replacement therapy and smoking, previous pregnancy, number of pregnancies, number of live births, breastfeeding, oral contraceptive use, or body mass index.

We examined a potential combined effect of hormone replacement therapy and past oral contraceptive use in post menopausal women compared to never exposed women by grouping women into three categories: exposure to both, exposure to one or the other and never exposure, and we also examined duration of exposure as never, up to ten years, and ten or more years. In each analysis, point estimates and associated confidence intervals were not appreciably different than for hormone replacement therapy alone (results not shown).

Results for either oral contraception or hormone replacement therapy were unchanged when the seven cases in whom proxies interviews were used and their matched controls were removed from the analyses (results not shown).

### Reproductive and related exposure categories

We evaluated several exposure categories related to reproduction and endogenous hormones, including age at menarche, number of live births, age at first live birth, history of breastfeeding and did not find significant associations with meningioma (Table 4). In addition we evaluated if reporting menstrual cycles or being post-menopausal on the reference date and also 10 years before the reference date were related to the risk of meningioma. In these analyses we used unconditional logistic regression, restricted to post-menopausal women who did not use HRT. The odds ratio for the risk of meningioma in women who were post-menopausal on the reference date was 3.1 (95% CI, 1.2 – 7.9) compared to women who were menstruating and was 0.6 (95%, CI, 0.2 – 1.9) in women who were post-menopausal ten years before the reference date even after adjusting for age and education.

We also assessed body mass index (BMI) grouped as under/normal weight (<25), overweight (25–30), and obese (≥30)[21]. Compared to under/normal weight women on the reference date, the risk of meningioma in overweight women was 1.2 (95% CI, 0.7 – 2.0) and was 1.5 (95% CI, 0.9 – 2.4) in obese women.

### Hormone receptor expression

Progesterone receptor (PR) expression was common with 62 (44%) of tumors having >75% of cells expressing PR, 35 (24%) of tumors having 25–75 percent of cells expressing PR, 33 (23%) of tumors having <25% of cells expressing PR, and 12 (8%) of tumors with no cells expressing PR. In contrast, estrogen receptor (ER) expression was uncommon with only 1 tumor having >75% cells expressing ER and 1 tumor with <25% of cells expressing ER. The remaining 140 tumors (98%) did not express ER. In exploratory analyses we focused on associations between exposure categories and progesterone receptor expression dichotomized as low (<25% of cells expressing PR) or high (25% or more expressing PR). We were unable to compare never, current and past oral contraceptives or hormone replacement therapy use to PR expression due to insufficient numbers, so we compared never and ever oral contraceptive or hormone replacement therapy exposure (Table 5). We found a strong association between oral contraceptive use and risk of a tumor expressing low, but not high, PR compared to controls. We also found a positive association between number of live births and risk of a tumor expressing low, but not high, PR compared to controls. For post-menopausal women, we did not observe evidence of an association between hormone replacement therapy and a tumor expressing low or high PR. While we found evidence for an association between meningioma and menstruating 10 years before the reference date, we did not find a difference in risk for cases whose tumors expressed low or high PR.

## Discussion

We used a population-based case-control study of risk factors for intracranial meningioma to assess hormonal exposures and the risk of subsequent meningioma in women. Overall, the associations between meningioma and oral contraceptives or hormone replacement therapy in this study were relatively weak. The direction of association was toward higher risk of meningioma among past or current oral contraceptive users, and among users of combined estrogen-progestin hormone supplements in menopause, while the direction was toward lower risk among women who used unopposed estrogen during menopause. However, confidence intervals around these odds ratios were wide and included one. While current post-menopausal status was associated with an increased risk of meningioma, post-menopausal status 10 years before the reference date had a non-significant protective effect. Exposures in this time frame are consistent with the theory of meningioma development where symptoms and diagnosis often occur 10 or more years after the initial establishment of the tumor[19].

The finding that meningioma risk is increased among women who were postmenopausal on the reference date, but decreased among women who were postmenopausal 10 years prior to the reference date is curious and could be due to chance given the number of comparisons made. However, a potential explanation is that the risk of developing meningioma may be associated with changing hormonal levels as opposed to the presence or absence of female sex hormones. The interval of transition from pre-menopausal hormone production to reduced production of endogenous hormones may be a risk factor for meningioma growth. Women who do not develop an apparent meningioma at this time pass through this higher risk window and enter a lower risk window.

The frequency with which meningioma tumors express estrogen receptors remains unclear with researchers reporting between 0 to 94% prevalence[6]. In this study only 2 of 142 specimens expressed estrogen receptors for a prevalence of 1.4%, whereas 130 tumors (92%) expressed progesterone receptors. The presence of progesterone receptor expression in meningioma tissue has been observed in previous pathology studies[14,16,22]. We were able to consider the combination of progesterone receptor expression and hormone exposure history. Others have observed that the grade of meningioma and likelihood of recurrence are related to progesterone receptor expression with tumors lacking such expression more likely to be higher grade and to recur[16,23] Results for history of live births were similar to those observed for oral contraceptives, with these exposures significantly or near significantly associated with tumors that have little or no PR expression. Hormone use could accelerate, retard or have no effect on growth of a meningioma. Whether growth is accelerated or retarded would depend on the specific biochemical pathways that are activated by the receptor when a hormone is present, whereas hormone use might plausibly have no effect on growth in tumors that do not express receptors. Though our study was not designed to assess this question, these results could be used to form a hypothesis for a study that seeks to determine whether progesterone receptor expression is related to several exposure categories, and to determine whether prognosis based on PR expression may differ based on endogenous and exogenous hormone exposures.

Our results suggesting little evidence for associations between reproductive and related exposure categories and meningioma are similar to those recently reported by Hatch and colleagues from a hospital-based case-control study of women consisting of 151 meningioma cases and 436 frequency-matched controls[17]. In this study, the point estimates of the odds ratios between meningioma and current or past use of OC were somewhat higher than those from the Nurses' Health Study cohort but the associations were not statistically significant in either study[18]. We did not find significant evidence of increased risk of meningioma in women who are past and current hormone replacement therapy users as reported by Jhawar and colleagues. We believe our ascertainment and pathological confirmation of meningioma for all cases and structured interview including visual prompts to ascertain hormone therapy exposure provide reliable data on disease and exposure history. However, interpretation of these results is difficult due to small numbers, unstable estimates, and inconsistencies with prior works.

This study cannot rule out a modest association between the risk of meningioma and body mass index as this finding is similar in both our study and that of Jhawar and colleagues. However, both height and weight were self-reported during the interview and may be subject to reporting error[24]. Our body mass index categories are consistent with those typically applied to the U.S. population[21,25,26]. Although Jhawar and colleagues used BMI categories consistent with the World Health Organization categories, which have lower cut-off values for overweight and obese groups, in both studies the same magnitude of increasing risk of meningioma was observed with increasing BMI.

This study has limitations. Chiefly, while disease status was confirmed, all exposures are self-reported, and we did not attempt to validate these exposures using medical or pharmacy records. Point estimates for several exposure categories suggest either elevated or reduced risk of meningioma, but the available sample sizes were not sufficient for specific combinations of exposures to suggest a coherent pattern of association. Furthermore, due to incomplete responses for oral contraceptive preparations we were unable to assess whether there is a different risk of meningioma resulting from opposed versus unopposed estrogen preparations. Analysis of all oral contraceptive preparations together could obscure an association between specific hormone preparations.

One potential limitation is related to the recruitment of controls. Random digit dialing was used to identify controls. In persons 65 years or older, random digit dialing was observed to be inefficient, so Medicare eligibility lists were used to identify controls. In each case our participation proportion was over 50% of the eligible controls. These participation proportions are consistent with those reported for other epidemiologic studies[27]. However, we cannot rule out that selection bias could play a role in our generally non-significant conditional logistic regression findings.

Another limitation is the potential for differential recall based on the elapsed time from reference date to the interview. However, we believe the potential for differential recall is more apt to be related to the nature of the reference date as opposed to time between the reference date and the interview date. For cases the reference date is the date of surgical resection of the meningioma, whereas for controls it is the date of surgery for the matched case, which is unlikely to have inherent meaning for the controls. To limit differential recall participants were asked to focus on specific time periods of interest beginning with exposure that would have been years before the reference date. The trained interviewers used memory priming techniques to facilitate recall of events before initiation of the interviews. For sex hormone and reproductive history, both current and remote exposure histories were assessed.

Although the presence of an association between meningioma and breast carcinoma remains uncertain, evidence of consistent findings for the role of specific exposures for both categories of neoplasm is mounting. Reports suggest the association between either oral contraceptive use or postmenopausal hormone use and risk of breast carcinoma may differ in relation to histopathology[28,29]. However, similar to reports for breast carcinoma, post-menopausal women who used unopposed estrogen (estrogen replacement therapy) had a either a reduced or no observed increase in the risk of meningioma whereas women who used opposed estrogen therapy had a modest increased risk of meningioma[10,30].

## Conclusion

The analyses of the association between meningioma and female sex hormones suggests that certain exposures may influence tumor biology and additional, larger studies that specifically assess whether hormone exposures and receptor expression are linked would help advance the understanding of why some women develop these relatively rare tumors while others do not. Overall the association between oral contraceptives or hormone replacement therapy and meningioma in women reported here is consistent with other studies and is not sufficiently evident to raise specific concerns about or suggest changes to current hormone therapy guidelines.

## Competing interests

The author(s) declare that they have no competing interests.

## Authors' contributions

WL, TK, GvB participated in the design of the study and acquisition of funding.

WL, BC participated in data collection.

LP, BC participated in data preparation and statistical analysis.

BC drafted the manuscript.

BC, WL, LP, TK, GvB provided interpretation of the results, critical revision and approval of the final version of the manuscript.

## Pre-publication history

The pre-publication history for this paper can be accessed here:



## Figures and Tables

**Table 1:** Demographic characteristics of women with intracranial meningioma and age and gender-matched controls

**Demographic Characteristic**	**Cases N = 143 Number (Percent)**	**Controls N = 286 Number (Percent)**
Age Group		
< 40	21 (15)	51 (18)
40–49	39 (27)	77 (27)
50–59	32 (22)	58 (20)
60–69	23 (16)	50 (18)
≥ 70	28 (20)	50 (18)
Race		
Caucasian	129 (90)	266 (93)
African American	2 (1)	7 (3)
Native American	3 (2)	4 (1)
Hispanic	2 (1)	4 (1)
Asian American	1 (1)	3 (1)
Other	6 (4)	2 (1)
Marital Status		
Single	15 (10)	34 (12)
Married	90 (62)	189 (66)
Separated/Divorced	16 (11)	33 (12)
Widowed	22 (15)	30 (11)
Educational Attainment		
Grade school	1 (1)	7 (3)
High school	54 (38)	72 (25)
Some college	74 (52)	165 (58)
Graduate school	14 (10)	42 (15)

**Table 2:** Association between oral contraceptive use and intracranial meningioma in women

Exposure*	**Cases n**	**Controls n**	**OR (95% CI)**†
Oral contraception (O C)			
Never	48	103	Referent
Past	90	176	1.5 (0.8 – 2.7)
Current use on reference date	5	7	2.5 (0.5 – 12.6)
Years of OC use			
Never	48	103	Referent
Up to 5 years	49	90	1.5 (0.8 – 3.0)
More than 5 years	46	93	1.4 (0.7 – 2.7)
First OC exposure relative to reference date			
Never	48	103	Referent
1–9 years before	7	15	1.6 (0.3 – 7.5)
10 or more years before	88	168	1.5 (0.8 – 2.7)

* Reported exposure before reference date, which for a case and her two matched controls was the date the case had surgery for her meningioma.

† Odds ratio (OR) and 95% confidence intervals (CI) estimated from multivariable conditional logistic regression including continuous age and three categories of education (high school or less, some college or trade school, and college graduate or graduate school) as confounding variables. None of the tests for linear trend was significant.

**Table 3:** Association between hormone replace therapy and intracranial meningioma in post-menopausal women

Exposure*	**Cases n**	**Controls n**	**OR (95% CI)**†
Hormone replacement therapy (HRT)			
Never	38	58	Referent
Past	40	73	0.7 (0.4 – 1.3)
Current use on reference date	23	41	1.0 (0.4 – 2.2)
Years of HRT use			
Never	38	58	Referent
Up to 5 years	28	43	0.9 (0.4 – 1.7)
More than 5 years	32	65	0.9 (0.5 – 1.7)
First HRT exposure relative to reference date			
Never	38	58	Referent
1–9 years before	30	44	0.9 (0.4 – 1.9)
10 or more years before	31	62	0.9 (0.5 – 1.6)
Type of HRT preparation			
None	38	58	Referent
Estrogen	33	66	0.9 (0.5 – 1.6)
Estrogen and Progestin	24	33	1.3 (0.6 – 2.8)

* Reported exposure before reference date, which for a case and her two matched controls was the date the case had surgery for her meningioma.

† Odds ratio (OR) and 95% confidence intervals (CI) estimated from multivariable conditional logistic regression including continuous age and three categories of education (high school or less, some college or trade school, and college graduate or graduate school) as confounding variables. None of the tests for linear trend was significant.

**Table 4:** Reproductive history and related endogenous exposures

Exposure*	**Cases n**	**Controls n**	**OR (95% CI)**†
Age at menarche			
< 12	22	44	Referent
12 – 14	100	212	0.9 (0.5 – 1.7)
> 14	21	30	1.2 (0.6 – 2.7)
Number of live births			
None	18	58	Referent
1	22	28	2.0 (0.9 – 4.6)
≥ 2	103	200	1.4 (0.7 – 2.6)
Age at first birth			
< 20	25	44	Referent
20 – 29	86	159	1.2 (0.6 – 2.4)
≥ 30	14	25	1.2 (0.4 – 3.8)
Ever Breast Feeding			
No	57	75	Referent
Yes	71	154	0.7 (0.4 – 1.0)
Menstrual status on reference date**			
Menstruating	21	72	Referent
Post-menopausal and not using HRT	38	58	2.8 (1.1 – 7.1)
Menstrual status 10 years before reference date**			
Menstruating	35	83	Referent
Post-menopausal and not using HRT	23	46	0.6 (0.2 – 1.9)

* Reported exposure before reference date, which for a case and her two matched controls was the date the case had surgery for her meningioma.

† Odds ratio (OR) and 95% confidence intervals (CI) estimated from multivariable conditional logistic regression including continuous age and three categories of education (high school or less, some college or trade school, and college graduate or graduate school) as confounding variables. None of the tests for linear trend was significant.

** Odds ratio (OR) and 95% confidence intervals (CI) estimated from multivariable unconditional logistic regression including continuous age and three categories of education (high school or less, some college or trade school, and college graduate or graduate school) as confounding variables.

**Table 5:** Association between exposure categories and progesterone receptor expression

**Explanatory Variable**	**Controls**	Cases with tumors expressing PR* in 0 to 25% of cells		Cases with tumors expressing PR* in = order 25 to 100% of cells	
	**n**	**n**	**OR (95% CI)**†	**n**	**OR (95% CI)**†

OC* use					
Never	103	15	Referent	33	Referent
Ever	183	31	3.2 (1.3 – 8.0)	64	1.1 (0.6 – 1.9)
HRT* use in post-menopausal women					
Never	58	15	Referent	23	Referent
Ever	114	22	0.8 (0.4 – 1.6)	41	0.9 (0.5 – 1.6)
Current menstrual status					
Menstruating	114	9	Referent	33	Referent
Post-menopausal	172	37	2.7 (1.2 – 5.8)	64	1.3 (0.8 – 2.1)
Menstrual status 10 years before reference date					
Menstruating	158	21	Referent	62	Referent
Post-menopausal	126	23	0.5 (0.2 – 1.5)	35	0.5 (0.2 – 1.0)
Current menstrual status					
Menstruating	72	4	Referent	17	Referent
Post-menopausal and not using HRT	58	15	2.4 (1.2 – 5.8)	23	3.1 (1.1 – 8.6)
Menstrual status 10 years before reference date					
Menstruating	83	8	Referent	27	Referent
Post-menopausal and not using HRT	46	10	0.3 (0.1 – 2.5)	13	0.8 (0.5 – 1.3)
Smoking status 10 years before reference date					
Non-smoker	144	29	Referent	48	Referent
Smoker	142	17	0.5 (0.3 – 1.0)	49	1.0 (0.6 – 1.5)
Body Mass Index on reference date					
< 25	156	23	Referent	45	Referent
25 – 30	66	12	1.2 (0.5 – 2.5)	22	1.1 (0.5 – 2.4)
≥ 30	64	11	1.1 (0.6 – 2.0)	29	1.5 (0.8 – 2.6)
Number of live births					
None	58	2	Referent	16	Referent
1	28	5	4.0 (0.7 – 22.3)	17	2.1 (0.9 – 4.9)
≥ 2	200	39	4.3 (1.0 – 18.8)	64	1.1 (0.6 – 2.2)

*PR, progesterone receptor; OC, oral contraceptive; HRT, hormone replacement therapy

† Odds ratio (OR) and 95% confidence intervals (CI) estimated from multivariable polytomous logistic regression including continuous age and three categories of education (high school or less, some college or trade school, and college graduate or graduate school) as confounding variables. None of the tests for linear trend was significant.
